# Advances in utilization of renewable substrates for biosurfactant production

**DOI:** 10.1186/2191-0855-1-5

**Published:** 2011-03-28

**Authors:** Randhir S Makkar, Swaranjit S Cameotra, Ibrahim M Banat

**Affiliations:** 1 279 Sweet Alyssum Dr. Ladson SC. 29456, USA; 2 Scientist F, Fellow AMI, FNABS, NESA Environmentalist, Member WFCC Task Groups, Institute of Microbial Technology, Sector 39A, Chandigarh-160036, India; 3 Professor Ibrahim M. Banat BSc PhD CBiol FIBiol, School of Biomedical Sciences, Faculty of Life and Health Sciences, University of Ulster, Coleraine BT52 1SA, Northern Ireland, UK

## Abstract

Biosurfactants are amphiphilic molecules that have both hydrophilic and hydrophobic moieties which partition preferentially at the interfaces such as liquid/liquid, gas/liquid or solid/liquid interfaces. Such characteristics enable emulsifying, foaming, detergency and dispersing properties. Their low toxicity and environmental friendly nature and the wide range of potential industrial applications in bioremediation, health care, oil and food processing industries makes them a highly sought after group of chemical compounds. Interest in them has also been encouraged because of the potential advantages they offer over their synthetic counterparts in many fields spanning environmental, food, biomedical, petrochemical and other industrial applications. Their large scale production and application however are currently restricted by the high cost of production and by the limited understanding of their interactions with cells and with the abiotic environment. In this paper, we review the current knowledge and latest advances in the search for cost effective renewable agro industrial alternative substrates for their production.

## Introduction

Surfactants are an important class of chemical products with high volume use in a great variety of household and industrial applications (Singh et al. 2007; Develter & Lauryssen, 2010; Franzetti et al., 2010). Their production was estimated in 2007 to be around 10 million tons per year. (Van Bogaert et al. 2007). Most of these surfactants are petroleum based and are chemically synthesized. However the leading trend towards using environmental friendly technologies has enhanced the search for biodegradable compounds of natural origin. Biosurfactants are therefore the natural choice for such processes as they possess a host of advantages over synthetic surfactants, such as lower toxicity, biodegradability and effectiveness at a wide range of pH and temperature values (Banat et al. 2010; Cameotra et al. 2010). Most biosurfactants, like synthetic surfactants, exhibit physicochemical properties and characteristics such detergency, emulsification, de-emulsification, foaming and wetting (Banat et al. 2000; Coimbra et al. 2009). These molecules have the abilities to reduce superficial and interfacial tension reduction between solids, liquids and gases. Interest in their potential applications by various industries has significantly increased recently, particularly because of their environmental friendly nature and sustainability (Banat et al. 2000; Cameotra and Makkar 2004; Mulligan 2009; Mulligan and Gibbs 2004; Van Hamme et al. 2006; Nitschke & Costa, 2007).

## Natural Choice for Bioremediation: Biosurfactants

**Figure 1:**
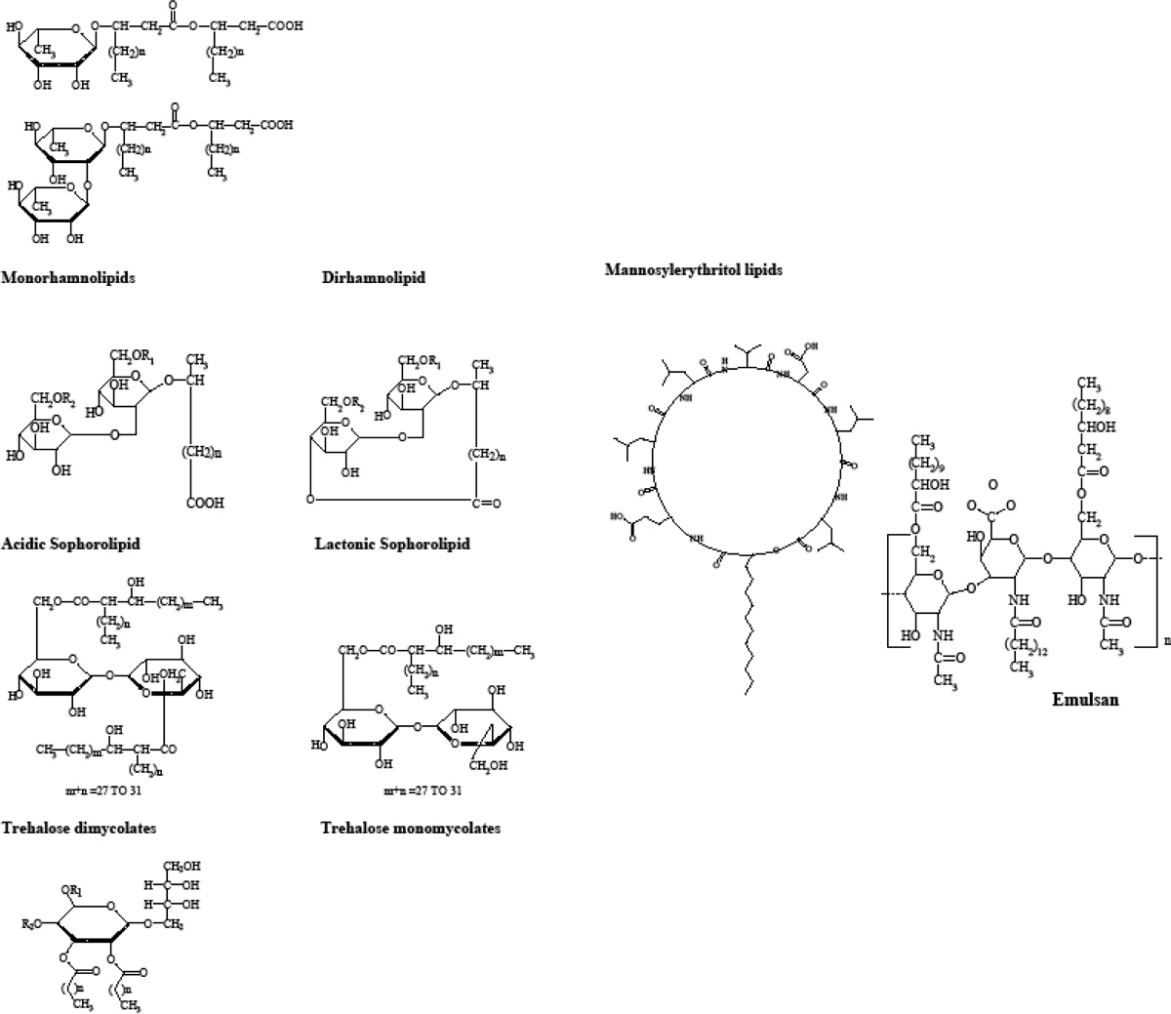
**a and b Representative Biosurfactants produced by microorganisms utilizing water soluble and/or water insoluble substrates**.

From an environmental standpoint, biosurfactants are more acceptable for the remediation process both at sea and land (Cameotra and Bollag 2003; Cameotra and Makkar 2010). They are structurally diverse and can have various chemical compositions mainly consisting of fatty acids, glycolipids, lipopeptides, lipopolysaccharides and lipoproteins depending on the producing microorganism, raw matter and process conditions. Various types are produced during microbial growth on water- immiscible substrates although not exclusively. This makes them more competitive and suitable to various application needs (Banat et al. 2000; Cameotra and Makkar 1998; Cameotra and Makkar 2004; Nitschke et al., 2005b; Singh et al. 2007; Van Hamme et al. 2006). Biosurfactants are classified based on their chemical structure and the organisms that produce them. Universally a typical biosurfactant is composed of hydrophilic component (mainly amino acids, peptide anions or cations, mono/disaccharides, polysaccharides) and a hydrophobic component (mainly saturated or unsaturated fatty acids) (Banat et al. 2000; Desai and Banat 1997; Smyth et al 2010a, b). Various types of biosurfactants, their structure and applications are represented in Table 1 and Figure 1a, b.

**Table 1:** Biosurfactants, producing organisms and their applications in recent years.

Organism	Type of biosurfactant	Potential Applications	Reference
*Rhodococcus erythropolis *3C-9	Glucolipid and a trehalose lipid	Oil spill cleanup operations	(Peng et al. 2007)
*Pseudomonas aeruginosa *S2	Rhamnolipid	Bioremediation of oil contaminated sites	(Chen et al. 2007)
*Pseudozyma siamensis *CBS 9960	Mannosylerythritol lipid	Promising yeast biosurfactant	(Morita et al. 2008a)
*Pseudozyma graminicola *CBS 10092	Mannosylerythritol Lipid	washing detergents	(Morita et al. 2008b)
*Pseudomonas libanensis *M9-3	Lipopeptide	Environmental and biomedical applications	(Saini et al. 2008)
*Bacillus subtilis *strain ZW-3	Lipopeptide	Potential in pharmaceutics, environmental protection, cosmetic, oil recovery	(Wang et al. 2008b)
*Rhodococcus *sp. TW53	Lipopeptide	Bioremediation of marine oil pollution.	(Peng et al. 2008)
*Pseudozyma hubeiensis*	Glycolipid	Bioremediation of marine oil pollution	(Fukuoka et al. 2008)
*R. wratislaviensis *BN38	Glycolipid	Bioremediation applications	(Tuleva et al. 2008)
*Bacillus subtilis *BS5	Lipopeptide	Bioremediation of hydrocarbon- contaminated sites	(Abdel-Mawgoud et al. 2008)
*Azotobacter chroococcum*	Lipopeptide	Environmental applications.	(Thavasi et al. 2008b)
*Pseudomonas aeruginosa *BS20	Rhamnolipid	Bioremediation of hydrocarbon- contaminated sites.	(Abdel-Mawgoud et al. 2009)
*Micrococcus luteus *BN56	Trehalose tetraester	Bioremediation of oil-contaminated environments.	(Tuleva et al. 2009)
*Bacillus subtilis *HOB2	Lipopeptide	Enhanced oil recovery, bioremediation of soil and marine environments, and food industries.	(Haddad et al. 2009)
*Pseudomonas aeruginosa *UFPEDA 614	Rhamnolipid	Bioremediation.	(Neto et al. 2009)
*Nocardiopsis alba *MSA10	Lipopeptide	Bioremediation	(Gandhimathi et al. 2009)
*Pseudoxanthomonas *sp. PNK-04	Rhamnolipid	Environmental applications.	(Nayak et al. 2009)
*Pseudozyma parantarctica*	Mannosylmannitol lipid,	Emulsifiers and/or washing detergents	(Morita et al. 2009)
*Pseudomonas alcaligenes*	Rhamnolipid	Environmental applications.	(Oliveira, et al.2009)
*Pseudomonas koreensis*	Lipopeptide	Biocontrol Agent	(Hultberg et al. 2010)
*Pseudomonas fluorescens *BD5	Lipopeptide	Bioremediation and biomedicine.	(Janek et al. 2010)
*Candida bombicola*	Sophorolipids	Environmental applications.	(Daverey and Pakshirajan 2010a, b)
*Brevibacterium *aureumMSA13	Lipopeptide	MEOR	(Kiran et al. 2010b)
*Nocardiopsis lucentensis *MSA04	Glycolipid	Bioremediation in the marine environment.	(Kiran, et al. 2010a)
*Bacillus velezensis *H3	Lipopeptide	Industrial strain for the Lipopeptide production.	(Liu et al. 2010)
*Calyptogena soyoae*	Mannosylerythritol lipid	Bioremediation processes in the marine environment.	(Konishi et al.2010)
*Burkholderia plantari *DSM 9509	Rhamnolipid	Detergents and pharmaceutical industry	(Hörmann, 2010)

In spite of their numerous advantages over the synthetic chemical surfactants the problem related with the large scale and cheap production still exits and is a major hurdle in economic competitiveness. This has led to concentrated efforts during the past decade, focused on minimizing production costs in order to facilitate wider commercial use.

## Economics of Biosurfactant Production

Economical large scale production for established and new applications of biosurfactants remains a challenge (Bognolo 1999). The biosurfactant surfactin (98% purity) available from Sigma Chemical Company costs approximately $153 for a 10 mg vial. An estimate by Ron and Rosenberg (Rosenberg and Ron 1997) for the cost of the RAG-1 emulsan containing broth was 50 dollar/kg and would therefore cost much more to extract, concentrate or purify the product. It is important to note however that their higher potency makes them better than commercial surfactant. In comparison the cost of chemical surfactants is around one dollar/lb http://www.purchasing.com/article/227703 however when taking into consideration the environmental damage they may cause, the cost ultimately becomes much more than a dollar. The perfect scenario would be to have biosurfactant priced in the range 3-5 dollars/lb. Improvement in production procedures and technologies has helped to some extent and can lead to further improvements.

Researchers have emphasized the key parameters affecting the efficiency of biosurfactant production in terms of higher yields and lower production costs (Bognolo 1999; Kosaric 1992; Mukherjee et al. 2006). According to them the biosurfactant formation and accumulation follows the basic facts of metabolic process and need to be studied comprehensively. According to (Syldatk and Hausmann 2010) the reasons for limited use of microbial surfactants in industry are the use of expensive substrates, limited product concentrations, low yields and formation of product mixtures rather than pure compounds. All these factors and other growth and upscale problems like use of antifoaming agents add on to the high costs of the downstream processing. The main strategy to achieve this are through (i) assessment of the substrate and product output with focus on appropriate organism, nutritional balance and the use of cheap or waste substrates to lower the initial raw material costs involved in the process; (ii) development of efficient bioprocesses, including optimization of the culture conditions and cost-effective separation processes to maximize recovery; and (iii) development and use of overproducing mutant or recombinant strains for enhanced yields (Bognolo 1999; Kosaric 1992; Mukherjee et al. 2006).

The use of the alternative substrates such as agro based industrial wastes is one of the attractive strategies for economical biosurfactants production. (Kosaric 1992) suggested the use of industrial and/or municipal waste waters, rich in organic pollutants, to achieve a double benefit of reducing the pollutants while producing useful products. Another approach involves using raw substrates with negligible or no value. However although this appears simple, the main problem associated with this approach is the selection of suitable waste material with the right balance of nutrients that permits cell growth and product accumulation. Another problem associated with this approach is the effects of the constituents on the properties of the final product. Although there are contrasting reports on this, some of the biosurfactants produced using agro-based substrates have similar structural and functional properties to those produced on synthetic media (Makkar and Cameotra 1997a; Makkar and Cameotra 2002). Another approach for reducing the production costs is developing processes which use renewable lowcost raw materials or high-pollutant wastes. A wide variety of alternative raw materials are currently available as nutrients for industrial fermentations, namely various agricultural and industrial byproducts and waste materials (da Silva et al. 2009; Deleu and Paquot 2004; Ferreira 2008; Makkar and Cameotra 2002; Montoneri et al. 2009a, b; Savarino et al. 2007).

Thus the promising future of biosurfactants appears to specifically depend upon the use of abundant and low-cost raw materials and the optimization of the operational cultivation conditions in order to achieve high yields. The development of low-cost processes and raw material can account for 10-30% of the final product cost. Further optimization of culture medium and growth conditions can significantly increase the yield (Cameotra and Makkar 1998; Mukherjee et al. 2006; Mukherjee et al. 2008; Mutalik et al. 2008). In addition the positive economical outlook can be enhanced by increasing their high throughput values or by harnessing other important properties such as pharmacological, antifungal and antiviral capabilities. Several biosurfactants have recently been used or anticipated to use in cost effective applications in medicine, food and cosmetic industries (Cameotra and Makkar 2004; Lourith and Kanlayavattanakul 2009; Mulligan 2009; Rodrigues et al. 2006a; Singh et al. 2007; Singh and Cameotra 2004)

It has been suggested that successful approaches to more economical production technologies of biosurfactant will be a collaborative approach involving process development and sustainable raw materials supplies. According to Smyth et al. (Smyth et al. 2010a, b) emphasis should be on cost effective management of downstream processing. The potential to obtain pure biosurfactants is dependent on several complex extraction and purification steps. Use of simple substrates with less downstream processing will economize the process and the use of agricultural substrates and their wastes represents a positive step towards achieving that goal. Some of the prevalent downstream processing process uses solvent extraction (e.g. chloroform-methanol, dichloromethane-methanol, butanol, ethyl acetate, pentane, hexane, acetic acid, ether) or acid precipitation, use of ammonium sulfate precipitation, crystallization, centrifugation, adsorption and foam fractionation (Chen et al. 2008; Kaar et al. 2009; Martins et al. 2006; Mukherjee et al. 2006; Neto et al. 2009). Use of statistical experimental strategies including factorial design and response surface methodology (RSM) will help in better optimization of solid state production of biosurfactants. Recently, (Kiran et al. (2010a) reported the production of a new glycolipid biosurfactant from marine *Nocardiopsis lucentensis *MSA04 in solid-state cultivation. More studies are needed on these processes for efficient production of biosurfactants. The availability of processes with limited downstream processing will give significant economical advantages and have been sought after.

## Potential substrates for biosurfactants' production

Plant biomass is an valuable resource to man and the value of the biomass contents is related to the chemical and physical properties of its molecules (Pérez et al. 2002). It is the main foreseeable sustainable source of organic fuels, chemicals and bio-materials, and significant efforts are made to make the 21^st ^century one that is based on renewable substrates. In addition the bioconversion of waste materials is considered to be of prime importance for the near future because of its favorable economics, low capital and energy cost, reduction in environmental pollution, and relative ease of operation (Deleu and Paquot 2004; Ferreira 2008; Montoneri et al. 2009a, b; Savarino et al. 2007). Producing usable products from agro industrial waste is therefore a feasible and favorable option (Makkar and Cameotra 2002; Moldes et al. 2007; Pandey et al. 2003).

Modern society produces high quantity of waste materials through activity related to industries, forestry, agriculture and municipalities (Martins et al. 2006; Montoneri et al. 2009a, b). The principal approaches of accumulating these wastes in landfills has resulted in several environmental problems including health related issues, increased safety hazards associated with gas generation and undermining sustainable development in terms of resource recovery and recycling of waste materials. Recently more thorough approach towards enhancing sustainability and resource recovery has influenced solid waste management practices. This practice, which is becoming norm in developed countries, is gradually gaining support in developing countries. Legal guidelines and constitutional directives to reduce waste generation and promote waste recovery have been laid down to encourage, reuse, recycling and energy recovery from waste materials. Wastes from urban activities and agro industrial practices are an important source of ligoncellulosic materials (Martins et al. 2006; Moldes et al. 2007; Montoneri et al. 2009a, b). The vast potential associated with these wastes has not yet been harnessed and is mostly desirable to release the pressure on space availability, shortage of agricultural crops suitable for human consumption and environmental concerns related to landfills use.

These inexpensive agro-industrial wastes substrates include Olive oil mill effluent, plant oil extracts and waste, distillery and whey wastes, potato process effluent and cassava wastewater. These waste materials are some examples of food industry byproducts or wastes that can be used as feedstock for biosurfactant production. The use of such waste materials serves a dual role, of generating a usable product and reducing waste disposal. This review is a compilation of literature for studies carried out exploring production of biosurfactants using different substrates developed mainly from renewable agro-industrial products.

## Biosurfactant Production Using Byproducts of Vegetable industries

Vegetable oils are a lipidic carbon source and are mostly comprised of saturated or unsaturated fatty acids with 16-18 carbon atoms chain. Researchers have used variety of vegetable oils from canola, corn, sunflower, safflower, olive, rapeseed, grape seed, palm, coconut, fish and soybean oil. The world production of oils and fats is about 2.5-3 millions tons, 75% of which are derived from plants and oil seeds (Dumont and Narine 2007). According to Haba et al. (Haba 2000), vegetable industries generate great amounts of wastes and their disposal is a serious problem. The crude or unrefined oils extracted from oilseeds are generally rich in free fatty acids, mono-, di-, and triacylglycerides, phosphatides, pigments, sterols, tocopherols, glycerol, hydrocarbons, vitamins, protein fragments, trace metals, glycolipids, pesticides, resinous and mucilaginous materials (Dumont and Narine 2007).

These agricultural based wastes are influenced by the agricultural practices and industries and are based in particular regions or countries. For example, in Brazil, the production of soap stock (one of the wastes of the oil neutralization process in soybean oil refining) amounts to 2-3% of the total oil production and is affected by the fatty acid content of the oil. Brazil is also among the main producers of vegetable oils, such as soybean oil, babassu oil and palm oil (Oliveira et al. 2009). India, another major developing country, has a high capacity to generate vegetable oil and there are significant waste from industries associated with soybean, sunflower, olive, groundnut, rapeseed, safflower, sesame, coconut, palm and mustard oils refining among many others (Pandey et al. 2003). The contribution of developed countries is also significant, in the United States for example, soybean oil refining processes potentially produce 100 million pounds of soap stock, which retails at 1/10th the cost of the refined vegetable oil (Dumont and Narine 2007). The raw waste and the wastewaters generated from these industries are major source of water and land pollution because of problems in degradability of high lipid components of these wastes (Cammarota and Freire 2006).

The enormous costs associated with treating these wastes using conventional treatment methods have been a major concern for the waste generators and responsible municipal authorities. The high content of fats, oils and other nutrients in these waste make them interesting and cheap raw materials for industries involved in useful secondary metabolite production. Conversion of this waste from the oil refining process to value added materials, presents a considerable challenge given the chemical complexity of these waste materials. This signifies the importance of developing more economically feasible chemical modification, identification and separation techniques.

A large body of literature on biosurfactant production using substrate related to vegetable industries exists and has a geographical significance in relation to industries associated and type of biosurfactant produced. The forthcoming sections in this review therefore are divided on the basis of type of the substrate related to vegetable oil or its wastes.

## Biosurfactant production using single substrate of Vegetable processing industries

(Mercade et al. 1993) were the first group to show the production of rhamnolipids by *P. aeruginosa *47T2 when grown on olive oil mill effluent (OOME) as the sole carbon source (a major waste problem in Spain). This study was important in demonstrating the possibility of using other lipophillic wastes for wider application. Kitamoto et al. (Kitamoto et al. 1993) studied the interfacial and antimicrobial properties of two kinds of mannosylerythritol lipids (MEL-A and B), biosurfactants, produced by *Candida antarctica *T-34, when grown on soybean oil as substrate. Since the biosurfactant produced in this study exhibited antimicrobial activity particularly against Gram-positive bacteria, the process could be more economical because of high value application in pharmaceutical industry. Sim et al. (Sim et al. 1997) have tested mixture of vegetable oils (canola oil, soy bean and glucose), for rhamnolipid production by *P. aeruginosa *UW-1 and reported 10-12 fold increase in rhamnolipid production on vegetable oils in comparison to glucose. Vollbrecht et al. (Vollbrecht et al. 1999) found the capability of *Tsukamurella *spec. (DSM 44370) growing on sunflower oil rich in oleic acid to produce a mixture of oligosaccharide lipids. They observed approximately 30 g/l glycolipid was produced from 110 g/l sunflower oil. The biosurfactant produced exhibited high surface and interfacial activity and some antimicrobial activities against some bacteria and a fungal strain which can contribute to the economical appeal of the process. (Mata-Sandoval and coworkers 1999) also report production of a mixture of rhamnolipid from *Pseudomonas aeruginosa *UG2 cultures grown on corn oil as sole carbon. (Camargo-de-Morais et al. 2003) studied the production of a glycolipid with emulsifier properties during cultivation of *Penicillium citrinum *on mineral medium with 1% olive oil as carbon source. The growth associated emulsifier production reached maximal activity at 60 h of cultivation with the production yield (Yp/s) of 0.54. Emulsifier which was stable in a wide range of pH and temperature was stimulated by high salt concentration implying a possible application in industrial waste or marine remediation. (Chang and coworkers 2005) reported the production of biosurfactant by *Pseudoxanthomonas kaohsiungensis *sp. nov. strain J36^T ^during cultivation on olive oil as the sole carbon and energy source.

(Thaniyavarn et al. 2006) examined the biosurfactant production by *Pseudomonas aeruginosa *A41, a strain isolated from seawater in the gulf of Thailand, grown in defined medium containing 2% vegetable oil or fatty acid as a carbon source. Culture medium exhibited excellent surface activity on each carbon source tested and biosurfactant production was observed even after the stationary phase. The type of carbon source affected the biosurfactant yield with maximum yield (6.58 g/l) was obtained with olive oil in comparison to palm oil (2.91 g/l) and coconut oil (2.93 g/l). Although, less yields was obtained with palm oil, the produced biosurfactant showed better surface activity and oil displacement values. This study demonstrated the possibility of having a low cost-large scale production of the microbial biosurfactant using palm oil as a cheap and abundantly available substrate. The biosurfactant produced had good surface activity and stability in wide ranges of pH, temperature and NaCl concentration, properties which allows possible uses and application in bioremediation and under wide range of conditions. In another study (Thaniyavarn et al. 2008) showed the sophorolipid production by *Pichia anomala *PY1, a thermo tolerant strain isolated from fermented food, using 4% soybean oil as carbon source at pH 5.5 at 30°C for 7 d. They observed the surface tension of the medium which decreased to 28 mN/m with crude oil displacement of 69.43 cm^2^.

(Rufino and co-workers 2007) studied the cultivation of *Candida lipolytica *grown on ground nut oil for production of a new biosurfactant. The preliminary investigation of chemical composition suggested it was a lipopeptide in nature. The biosurfactant had a yield of 4.5 g/l and exhibited good surface activity, emulsification ability and can withstand high salt concentration but was not thermo stable. They later also applied sequential factorial design to optimize biosurfactant production by *Candida lipolytica *using soybean oil refinery residue as substrate (Rufino et al. 2008). In this study they evaluated the impact of three cultivation factors, amounts of refinery residue, glutamic acid and yeast extract. The biosurfactant product showed high surface activity and emulsifying ability and was very stable at wide range of pH (2-12), temperatures (0-120°C) and salinity (2-10% NaCl). They concluded that combination of an industrial waste and a cheap substrate is a promising approach to reduce production cost. In another study the same group (Sobrinho et al. 2008) described a low cost medium for the production of a surfactant by the yeast *Candida sphaerica*. The medium formulation consisted of distilled water containing 5.0% groundnut oil refinery residue plus 2.5% corn steep liquor as substrates. The biosurfactant product had high surface tension reducing activity (26 mN/m), a low CMC value (0.08%) and a yield of 4.5 gl^-1^. The biosurfactant characterized was an anionic glycolipid (consisting of 75% lipid and 25% carbohydrate) and was stable at wide temperature, pH and salt level. They concluded that it was possible to produce biosurfactants from agricultural materials and use them in potential application in oil recovery from sand. (Coimbra et al. 2009) also showed the biosurfactant production by six *Candida *strains cultivated in insoluble (n-hexadecane) and soluble substrates (soybean oil, ground-nut oil refinery residue, corn steep liquor and glucose). These biosurfactant were able to remove 90% of the hydrophobic contaminants from sand.

Oliveira et al. (Oliveira et al. 2009) used palm oil, a low-cost agricultural byproduct which is used in as raw material for soap and food industries, for biosurfactant production using *Pseudomonas alcaligenes *(a strain isolated from crude oil contaminated soil). They achieved a biosurfactants concentration of 2.3 g/l and E_24 _more than 70% with the hexane, jet fuel and crude-oil. (Abouseoud et al. 2008) studied the production of a biosurfactant by *Pseudomonas fluorescens *Migula 1895-DSMZ and reported highest yield of rhamnolipid biosurfactant with olive oil and ammonium nitrate as carbon and nitrogen sources at a C: N ratio of 10. The biosurfactant exhibited good surface activity, emulsifying ability and stability at high temperature, salt concentration and wide range of pH in addition to some antimicrobial activity which gives it additional favorable application related properties. (Pornsunthorntawee and co-workers 2008) used *Bacillus subtilis *PT2 and *Pseudomonas aeruginosa *SP4, for biosurfactant production using a nutrient broth with palm oil as the carbon source. The optimum growth for the organisms was achieved in approximately 48 h and biosurfactant produced exhibited good surface activity. Comparative study of these biosurfactants with three synthetic surfactants showed better oil recovery efficiency by both biosurfactants.

(Abdel-Mawgoud et al. 2009) reported the production of a rhamnolipid by *Pseudomonas aeruginosa *isolate Bs20 on soybean oil amended medium. The rhamnolipid produced was s viscous sticky oily yellowish brown liquid with a fruity odor with a very high surface activity, emulsifying capacity and thermo and halo tolerance properties. These characteristics indicated that rhamnolipids were potential candidate for use in bioremediation of hydrocarbon-contaminated sites or in the petroleum industry a conclusion that was also confirmed by (Perfumo et al 2010). (Monteiro et al. 2009) reported the growth and biosurfactant production using sunflower oil supplemented mineral medium by the yeast *Trichosporon montevideense*, CLOA 72. The glycolipid produced exhibited good surface and emulsifying activity with vegetable oils, toluene, kerosene, isooctane, cyclohexane, hexane, diesel oil and hexadecane. The biosurfactant was thermo tolerant, halo tolerant and stable in wide range of pH values.

## Biosurfactant production using mixed substrates of Vegetable industries

To make processes more economical some researchers followed an approach of mixed substrates as carried out by (Casas and Garcia-Ochoa 1999) who, utilized the capability of *Candida bombicola *to produce sophorolipid biosurfactant properties when grown in medium composed of two different carbon sources and a nitrogen source. One of the carbon sources was a readily available sugar to maximize biomass production and the second was sunflower oil and they were able to achieve 120 g/l sophorolipid in 8 days under the best operational conditions. Haba (Haba et al. 2000) found nine *Pseudomonas *strains and two *Bacillus *strains capable of lowering the surface tension (to around 32-36 mN/m) and making stable emulsions with kerosene oil. Strain *Pseudomonas aeruginosa *47T2 produced 2.7 g/l of rhamnolipid with a production yield of 0.34 g/g with waste frying cooking oil (sunflower and olive oil) as substrates.

In an effort to economize biosurfactant production (Rau et al. 2001) used oleic acid or rapeseed oil respectively, as additional carbon sources in addition to glucose in an optimized feed-batch and continuous cultivations. They obtained high yields >300 g/l sophorolipid and increased productivities of 57 g/l/d (feed-batch) and 76 g/l/d (continuous mode), respectively, by using optimized cultivation conditions. (Rahman et al. 2002a, b) carried a study aimed at the development of economical methods for higher yields of biosurfactant by using low-cost raw materials. They achieved yields of 4.31, 2.98, and 1.77 g/l rhamnolipid biosurfactant using soybean oil, safflower oil, and glycerol, respectively by oil-degrading strain, *Pseudomonas aeruginosa *DS10-129. Trummler (Trummler et al. 2003) developed a biotechnological process for production of rhamnolipids by *Pseudomonas *sp. DSM 2874 using rapeseed oil as substrate. A yield of 45 g/l of mixtures of mono and di-rhamnolipids was obtained. Enzymatic modification of substrate by direct addition of Naringinase to resting cells resulted in production of rhamnolipid (1 - 4), L-(+) rhamnose and (*R*, *R*)-3- (3-hydroxydecanoyloxy) decanoic acid. This was one of the first attempts for an integrated microbial/enzymatic process for production of pure rhamnolipid.

(Bednarski et al. 2004) reported the synthesis of biosurfactants by *Candida antarctica *or *Candida apicola *in the cultivation medium supplemented with oil refinery waste (either with soap stock or post-refinery fatty acids). Enrichment of medium with the oil refinery waste resulted in a 7.5-8.5-fold greater concentration of glycolipids in comparison to the medium without addition of oil refinery waste. (Reis et al. 2004) investigated the production of biosurfactant by *Bacillus subtilis *ATCC 6633 using commercial sugar, sugarcane juice and cane molasses, sugarcane juice alcohol stillage, glycerol, mannitol, and soybean oil. Lower surface tension and higher emulsification indexes were achieved, indicating the feasibility to produce biosurfactants from a renewable and low-cost carbon source.

Costa (Costa et al. 2006) evaluated the possible use of oil from Buriti (*Mauritia flexuosa*), Cupuaçu (*Theobroma grandiflora*), Passion Fruit (*Passiflora alata*), Andiroba (*Carapa guianensis*), Brazilian Nut (*Bertholletia excelsa*) and Babassu (*Orbignya *spp.) for rhamnolipid production by *Pseudomonas aeruginosa *LBI. They observed extensive surface tension reduction and good emulsification. The highest rhamnolipid concentrations were obtained from Brazilian Nut (9.9 gl^-1^) and Passion Fruit (9.2 g/l) oils. Another Brazilian group led by Prieto (Prieto et al. 2008) isolated *P. aeruginosa *LBM10 from a southern coastal zone in Brazil, which could produce a rhamnolipid-type biosurfactant growing on different cheap carbon sources, such as soybean oil, soybean oil soapstock, fish oil and glycerol. Maximum yield was achieved with soybean oil as substrate and the biosurfactant was stable at a wide range of pH and salt concentration making it suitable for the bioremediation of spills in marine and estuarine environments.

A combination of sugarcane molasses and three different oils, (soybean, sunflower or olive oil) was used a low cost media by (Daverey and Pakshirajan 2009), for the production of sophorolipids (SLs) from the yeast *Candida bombicola*. They achieved a yield approx. 24 g/l in this mixed media in comparison to media with single constituents. This yield was comparable to the costly conventional synthetic medium containing yeast extract, urea, soybean oil and glucose. In their continuing work they studied the effect of various parameters on sophorolipid (SL) production by the yeast *Candida bombicola*. They achieved a yield of 60 g/l in the fermentor under optimal conditions defined as sugarcane molasses 50 g/l, soybean oil 50 g/l, inoculum size 5% (v/v), temperature 30°C, inoculum age 2 days, and agitation 200 rpm (Daverey and Pakshirajan 2010a). These studies along with model fitting in the work suggest that conventional medium containing glucose can very well be replaced with the present low-cost fermentative medium.

(Stoimenova et al. 2009) investigated the production of glycolipids biosurfactant by *Pseudomonas fluorescens*, from a variety of carbon sources, including hydrophilic compounds, hydrocarbons, mineral oils, and vegetable oils. They reported increased cell hydrophobicity was directly correlated with biomass increase indicating the presence of a mechanism based on interfacial uptake of hydrophobic substrates. In addition they concluded that stationary phase enhanced biosurfactant capability of *P. fluorescens *strain HW-6 to grow and utilize different nutrients as energy which may make it a promising candidate for its possible application in bioremediation.

Apart from studies where lipidic vegetable oils alone or mixed with other vegetable oils or other carbon source researchers were looking at more economic process of using wastes related to these industries.

## Biosurfactant production from Vegetable oil industries' wastes

(Benincasa et al. 2002) reported isolating a rhamnolipids producing *Pseudomonas aeruginosa *strain LBI using soap stock as the sole carbon source. Soap stock is the waste from the sunflower oil process, the main co-product from the seed-oil refining industry. Rhamnolipids concentration in range of 15.9 g/l was achieved. (Nitschke et al. 2005a) evaluated the oil wastes as an alternative low-cost substrates for the production of rhamnolipids by *Pseudomonas aeruginosa *LBI strain. They used wastes obtained from soybean, cottonseed, babassu, palm, and corn oil refinery. The soybean soap stock waste was the best substrate, generating 11.7 g/l of rhamnolipids and a production yield of 75%. The study is an evidence of the fact low cost substrate can be utilized for rhamnolipid production for application in high value pharmaceutical and food industry applications(Nitschke et al. 2010).

Another soybean-associated waste, which has been utilized for biosurfactant production, is Soy molasses, a byproduct of soybean oil processing. It contains high fermentable carbohydrate (30% w/v) and is about 60% of solids carbohydrate which makes it well suited for economical production of biosurfactants. Increased interest in consumption of healthy soy protein based foods and drinks have established a sustained growing soy based industry and as a result an abundance of waste byproducts (Deak and Johnson 2006). Soy molasses were used to produce sophorolipids by *Candida bombicola *(Solaiman et al. 2004). The yield of pure biosurfactant was 21 g/l. Recently the same group has shown that soy molasses act as carbon and nitrogen source for the fermentative production of sophorolipids by *Candida bombicola *with yields of 55 g/l (Solaiman et al. 2007). In this study they achieved a further cost reduction by substitution of expensive yeast extract and urea from the growth medium. The study opened a new frontier for applicability of low cost carbon reach substrates as a combined source of carbon and nitrogen for other industrial bioprocesses. The ability of *Candida bombicola *ATCC 22214 to produce sophorolipids using Turkish corn oil and honey was also investigated (Pekin et al. 2005). Biosurfactants was produced with both substrates with higher yield when corn oil and glucose were used. The scale up study was carried out in a 3 L bioreactor. Adapted culture on glucose and corn oil were supplemented with cheap market honey as the sole carbon source. A yield of about 400 g/l of sophorolipids was obtained.

In an effort to economize biosurfactant production (Thavasi et al. 2008a) used a mixture of peanut oil cake and waste motor lubricant oil as a substrate for the biosurfactant production. Peanut oil cake a rich source of carbohydrate, protein and lipids is a byproduct during the peanut oil manufacturing. The cost of peanut cake is negligible compared to other pure carbon sources and waste motor oil is a waste product generated by the geared motor vehicles' after long use. They confirmed that *Bacillus megaterium, Azotobacter chroococcum *and *Corynebacterium kutscheri *had the capability of using these substrates for biosurfactant production with better yields achieved with peanut oil cake. Recently the authors have reported the biosurfactant production by *Lactobacillus delbrueckii *using peanut oil cake as the carbon source. The biosurfactant produced (5.35 mg/ml) was capable of promoting biodegradation to a large extent reported (Thavasi et al. 2011). These studies showed the suitability of peanut oil cake as a substrate for glycolipid biosynthesis.

## Biosurfactant production using restaurant frying oil wastes

Another raw materials associated with vegetable industry is residual cooking or frying oil which is a major source of nutrient rich low cost fermentative waste. Large quantities of cooking oil are generated in restaurants worldwide. It has been estimated that on average 100 billion L oil waste/week is produced in United States alone (Shah et al. 2007). There are few reports, which utilized the vast potential of these frying oils for biosurfactant production.

(Fleurackers 2006) has shown *Candida bombicola *ATCC 22214 can produce biosurfactants in shake flask using restaurant oil waste. This was a successful feasibility study for waste frying oils as substrate. (Shah et al. 2007) studied sophorolipid production by *C. bombicola *in both batch and fed batch fermentations. They achieved a yield of 34 g/l sophorolipids on restaurant oil waste while (Zhu et al. 2007) achieved 20 g/l rhamnolipid using *Pseudomonas aeruginosa *zju.u1 M. a 50 L bioreactor. These studies demonstrated the feasibility to reusing waste frying oil for both sophorolipids and rhamnolipids production on industrial scale. (Sadouk et al 2008) in an approach for reducing the cost of production of glycolipids by *Rhodococcus erythropolis *16 LM.USTHB converted residual sunflower frying oil, a cheap renewable substrate, into extracellular glycolipids. With substrate concentration of 3% they could achieve high surface activity and emulsification capability from the biosurfactant. Their projected potential application included cleanup of hydrocarbons contaminated sites and for enhanced oil recovery. (de Lima et al. 2009) investigated the efficiency and magnitude of biosurfactant production by the *Pseudomonas aeruginosa *PACL strain using different waste frying soybean oils. The submerged cultivation process in stirred tank reactors of 6 and 10 liter capacities were carried out using a complete factorial experimental design, with the aim of optimizing the aeration rate and agitation speed. Aeration was identified as the primary variable affecting the process and at optimum levels; a maximum rhamnose concentration of 3.3 g/l, an emulsification index of 100% and a minimum surface tension of 26.0 mN/m was achieved. The waste soybean oils resulted in biosurfactant production of 75-90% of the maximum value, which was achieved when fresh soybean oil was used. The concluded that their strain has the potential to produce biosurfactant from waste frying soybean oil at low aeration rates. In search for cost effective biosurfactant production (Liu et al. 2009) compared the production of biodemulsifier by *Dietzia *sp. S-JS-1 using waste frying oil and paraffin as carbon source. The bioemulsifier produced on waste frying oil was better than the one produced on paraffin oil in terms of emulsifying capability. Ruggeri et al. (Ruggeri et al. 2009) isolated *Rhodococcus *sp. BS32 able to produce extracellular biosurfactants growing on rapeseed oil.

The studies mentioned above emphasize the potential application of vegetable oil and related substrates for the biosurfactant production. The use of vegetable and their byproducts/wastes as a source of biosurfactants and other functional compounds is promising, but requires a consolidated interdisciplinary efforts and research to full realization.

## Biosurfactant Production from Dairy and Sugar Industry wastes

The dairy industry has a considerable amount of by-products such as buttermilk, whey, and their derivatives. Whey is a liquid by-product of cheese production, rich in lactose (75% of dry matter) and containing other organic water-soluble components (12-14% protein). Whey has a high BOD value and its disposal can be problematic especially for countries depending on dairy economy. Only 50% of the cheese whey produced annually is recycled into useful products such as food ingredients and animal feed and the rest is regarded as a waste. (Daniel et al. 1998a) reported the high yields of sophorolipids production with whey concentrate and rapeseed oil as substrate. However in this study the organisms did not utilize lactose. In another study (Daniel et al. 1998b) demonstrated the production of high concentrations of sophorolipids (about 422 g/l) in a two stage cultivation process. In the first stage, deproteinized whey concentrate rich in lactose was used for the cultivation of *Cryptococcus curvatus *ATCC 20509. In the second stage, biomass obtained from first stage was homogenized under high pressure and autoclaved to generate a crude cell extract consisting of single cell oil which was used as substrate for growth of *C. bombicola *ATCC 22214 for sophorolipid production. (Daverey and Pakshirajan 2010b) also reported the production of sophorolipids by the yeast *Candida bombicola *on medium containing mixed hydrophilic substrate (deproteinized whey and glucose), yeast extract and oleic acid. They could achieve a yield up to 34 g/l under experimental conditions in optimized medium formulation.

Molasses are a co-product of sugar production, both from sugar cane and sugar beet industry in India as a runoff syrup from the final step of sugar crystallization after which further sugar crystallization becomes uneconomical (Maneerat 2005a). The main reasons for widespread use of molasses as substrate are its low price compared to other sources of sugar and the presence of several other compounds and vitamins (Makkar and Cameotra 1997b). Molasses are mainly composed of sugars (sucrose 48-56%), non sugar organic matter (9-12%), proteins, inorganic components and vitamins. The total fermentable sugar are in the range of 50-55% by weight. Traditionally molasses are used as an animal feed, production of pullulan, xanthan gum, citric acid and in ethanol industries (Maneerat 2005b).

Molasses with its high sugar content is a good substrate for biosurfactant production as evidenced by many studies covering two decades. Ghurye et al. (1994) were the first to report on non-aseptic production of biosurfactant from molasses by a mixed culture in stirred batch reactors. Biosurfactant production was directly correlated with biomass production, and was improved by pH control and addition of yeast extract. Molases were also used in growth and biosurfactant production using two strains of *Bacillus subtilis *(MTCC 2423 and MTCC1427) (Makkar and Cameotra 1997b). The surface activity and high emulsification index of biosurfactants indicated their potential application in microbial enhanced oil recovery. (Patel and Desai 1997) reported the production of rhamnolipid biosurfactants during growth on molasses and corn steep liquor as the primary carbon and nitrogen sources by a *Pseudomonas aeruginosa *GS3. The product had good surface activity and emulsification values with potential application in oil recovery.

The possibility of using soy molasses a relatively inexpensive and easily available resources to produce rhamnolipids was investigated by (Rashedi et al. 2005). They reported that biosurfactant production by the bacterial strain on soy molasses was growth related. The specific production rate of rhamnolipid when using 2%, 4%, 6%, 8% and 10% of molasses were 0.00065, 4.556, 8.94, 8.85, and 9.09, with rhamnolipids/biomass yield of 0.003, 0.009, 0.053, 0.041 and 0.213 respectively. Others such as (Raza et al. 2007) reported the production of a microbial surfactant by growing *Pseudomonas aeruginosa *EBN-8 mutant on clarified blackstrap molasses as a sole carbon and energy source. Maximum rhamnolipid (1.45 g/l) yields were observed, at 96 h of incubation on 2% total sugars-based molasses amended with sodium nitrate. In addition rhamnolipid biosurfactant production using eighteen strains of *Pseudomonas *sp. were investigated by (Onbasli and Aslim 2009). The two strains with highest yield of rhamnolipids production (*Pseudomonas luteola *B17 and *Pseudomonas putida *B12) were further examined for rhamnolipid production on different sugar beet molasses concentrations. Maximum rhamnolipid production was achieved with 5% (w/v) of molasses and occurred after 12 h incubation. There are also reports on use of molasses in combination with other substrates. (Dubey and Juwarkar 2001) investigated this possibility using *Pseudomonas aeruginosa *strain BS2 for the biosurfactant production from distillery and whey wastes and achieved of 0.97 g/l growth associated product.

Another important distillery waste produced in large volumes is the spent wash generated from alcohol distilleries. Increased demand for alcohol for applications in pharmaceuticals, food, perfumery industries and recently as an alternate fuel has increased the amounts generated of this waste (Mohana et al. 2009). (Babu et al. 1996) carried some batch kinetic studies on rhamnolipid biosurfactant production comparing synthetic medium, industrial wastes in comparison to distillery and whey waste as substrates. Their results show that *Pseudomonas aeruginosa *strain BS2 had better specific growth rates on both the distillery and whey wastes in comparison with the synthetic media. This study is significant in showing that both distillery and whey industrial wastes can be successfully utilized as substrates for biosurfactant production.

In search for potential alternative fermentative medium for biosurfactant production from *Lactococcus lactis *53 and *Streptococcus thermophilus *(Rodrigues et al. 2006b) used cheese whey and molasses. They reported an increase of 1.2-1.5 times in the mass of produced biosurfactant per gram cell dry weight with 75% cost reduction. They concluded that supplemented cheese whey and molasses media can be used as a relatively inexpensive and economical alternative to synthetic media for biosurfactant production by these probiotic bacteria. In a similar study biosurfactant production by *Bacillus licheniformis *K51, *B. subtilis *20B, *B. subtilis *R1 and *Bacillus *strain HS3 using molasses or cheese whey as a sole source of nutrition under thermophilic conditions higher yields were obtained with molasses at 5.0-7.0% (w/v) (Joshi et al. 2008). The biosurfactant obtained were both heat and pH stable and showed 25-33% recovery of residual oil through mobilization in the sand pack columns.

**I**n an effort to reduce the cost of surfactin production by *Bacillus subtilis *BS5 (Abdel-Mawgoud et al. 2008) optimized the environmental and nutritional production conditions for economizing of the production process. Optimized medium containing 16% molasses, 5 g/l NaNO_3 _and the trace elements solution of ZnSO4·7H_2_O (0.16 g/l), FeCl_3_·6H_2_O (0.27 g/l), and MnSO_4_·H_2_O (0.017 g/l) gave surfactin yield of 1.12 g/l.

In conclusion both molasses and whey has been successfully utilized as substrate for biosurfactant production. More studies however are required to overcome the problems associated with batch variability and ways to standardize the pre treatment requirement of these substrates for more productive output.

## Biosurfactant Production from Ligoncellulosic waste

Lignocellulosic materials are among the most abundant organic carbon available on earth (Kukhar 2009) and they are the major components of different waste streams from various industries, forestry, agriculture and municipalities. Such waste materials are mostly burned releasing CO_2 _which contributes to the greenhouse effect

Lignocellulose consists of mainly three types of polymers - cellulose, hemicellulose, and lignin that are strongly intermeshed and chemically bonded by both non-covalent forces and covalent cross-linkages. Microbial degradation of these macromolecules by fungi and bacteria has been extensively studied (Pérez et al., 2002). Such degraders usually utilize a battery of hydrolytic or oxidative enzymes to achieve this which is the subject of intensive research in many laboratories around the world. Usually they have been utilized as raw material for the production of ethanol and organic acids and these process has been reported to be economically feasible (Taherzadeh and K K 2007).

From an economical point of view, ligoncellulosic rich agricultural residues can be employed for producing useful biomolecules such as biosurfactants. There have been some reports of some forms of ligoncellulosic wastes for production of biosurfactant. (Moldes and coworkers 2001) have reported use of ligoncellulosic materials as substrates for production of lactic acid using lactobacilli strains (Bustos 2005; Moldes et al. 2001; Sreenath et al. 2001). Apart from this there are reports on production of biosurfactants by *Lactobacilli *on synthetic medium (Rodrigues et al. 2006c; Velraeds et al. 1996). (Portilla-Rivera et al. 2007a) were the first to look in to the capability of *Lactobacillus *sp to use hemicellulosic hydrolyzates from various agricultural residues for simultaneous production of biosurfactants and lactic acid. Such dual production strategy makes biosurfactant more economical viable in market and reduce the effects of waste burning on environment. In their efforts they achieved reduced surface tension and biosurfactant yield of 0.71 g/g of biomass, when hemicellulosic hydrolyzates form trimming vine shoots was used (Portilla-Rivera et al. 2007a). This study is important considering the large amount of pruning wastes of vine-stocks generated worldwide and the resulting constitutive monomeric sugar solutions, which are potential renewable sources for the other biomolecules like lactic acid. They concluded that hemicellulosic sugars from the agricultural residues are interesting substrates for the competitive cost production of biosurfactants.

In a further study by the same group (Portilla-Rivera et al. 2007b) utilized distilled grape marc another abundant by product of wine industry in Spain which consists of complex ligoncellulosic material. In viticulture, a huge amount of grape marc is produced after pressing the crushing grapes during wine making. Some of this grape marc is usually distilled in winery's to recover ethanol to be used in production of spirituous liquors. However, huge amounts of distilled grape marc remain unutilized marc which has huge amount of hemicelluloses and organic acids and can be utilized for production of useful biomolecules such as lactic acid and biosurfactants. In their effort to make biosurfactant production more economic Portllia-Rivera (Portilla-Rivera et al. 2008a) evaluated the sugars-containing liquors from hydrolyzates of distilled grape marc as media for the lactic acid and biosurfactants production. The study took into account the effects of variables temperatures, reaction times and H_2_SO_4 _concentration on hydrolysis using an incomplete factorial design. They reported a yield of 4.8 mg/l of intracellular biosurfactants, which is equivalent to 0.60 mg of intracellular biosurfactant per gram of sugar using *L. pentosus*. Further stability and emulsifying capacity studies on the obtained biosurfactants (Portilla-Rivera et al. 2008b) showed much better and stable emulsion volume (EV) than with commercial surfactin and other surfactants. This work demonstrates the possibility of using low cost agricultural residues as substrates for biosurfactant production with its economical implications.

In today's society increased urban activities generates vast amounts municipal solid wastes (MSW) which is a rich source of energy due to their high organic matter content and unfortunately mainly ends up in landfills. However space demand for growth of urban populations and the shrinking space available for these landfills and increasing environmental regulations is prompting research into alternative waste utilization technologies. (Montoneri et al. 2009a) reported one such process of converting these wastes to useful molecules' like biosurfactants or biophotosensitizer for diversified chemical applications. The results of this study shows that the biomass wastes can be an interesting source of chemical market. The biosurfactants produced using these biomass conversion processes offers a promising economic return which can be applied to gain some energy savings over synthetic surfactant production to enhance net profits.

## Biosurfactant from starch rich substrates

Starch is a major agricultural product of corn, tapioca, wheat and potatoes which are major crops. Other sources include sugar plants such as sugar beet, sugar cane or sugar sorghum. Sugar and starch processing industries also produce large amount of solid residues of starch containing wastewater. The high fiber content of the solid residue makes them a good source for paper and packaging industries. While the carbohydrates rich wastewater are a suitable substrate for production of microbial products. Biological wastes rich in starchy materials are suitable for biosurfactant production. These substrates have been feedstock for production of industrial enzymes and other related chemicals (Maneerat 2005a, b). One such substrate is potato which is one of the important staple food and a lucrative cash crop in many countries. Processing of potatoes, results in starch rich waste water, potatoes peels, un- consumable potatoes, which are rich substrates for the microbes. It is estimated that only 59% of the potato crop are processed into consumable products and most what remains represent a starchy rich wastes which can be difficult to dispose of. Conventional disposal methods include using as an irrigation source, as animal feed, or as a substrate for alcohol production (Fox and Bala 2000; Natu et al. 1991). (Fox and Bala 2000) attempted to produce biosurfactants utilizing potato associated waste. They evaluated potato substrate as a carbon source for biosurfactant production using *B. subtilis *ATCC 21332. They compared growth, surface activity and carbohydrate utilization of *B. subtilis *ATCC 21332 on an established potato medium, simulated liquid and solid potato waste media and a commercially prepared potato starch in a mineral salts medium. The results obtained indicated the utilization of potato substrate and production of surfactant as indicated by high surface tension reduction.

In their continuing work for reducing the process cost for biosurfactant they tested two types of potato process effluents waste, the high-solids (HS) and low-solids (LS) (Thompson et al. 2000). Although they obtained very low yield with these substrates compared to optimized potato starch medium they concluded that LS can be used for surfactin production for low-value applications such as environmental remediation or oil recovery (Thompson et al. 2000, 2001). Subsequently to improve the process for utilization of potato related substrate the same group integrated inexpensive substrate with the better downstream process of foam fractionation in an airlift reactor. They achieved limited successes in the process as it was restricted by the oxygen availability and competition for indigenous bacterial population (Noah et al. 2002). Their improvisation with the cultivation conditions and product recovery increased the surfactant yield to 0.6 g/l in about 2 days from the potato process effluents (Noah et al. 2005).

The efficiency of two *Bacillus subtilis *strains for the production of biosurfactants in two fermentation systems using powdered potato peels as substrate were investigated (Das and Mukherjee 2007). Potato peels were immersed in very hot water followed by oven drying. The dried peels were grinded to a paste and stored at 4°C before further use. Both the fermentation process resulted in biosurfactant (lipopeptides) with good surface activity and yield. (Wang et al. 2008a) applied a *Bacillus subtilis *strain B6-1, for production of biosurfactant using soybean and sweet potato residues in solid-state fermentation.

Another starch rich substrate with huge application for production of biosurfactants is cassava wastewater which is a carbohydrate rich residue (from the pressing of cassava roots) to obtain cassava flour, a common ingredient used in Brasilian cookery. Major nutrients present on cassava waste are sugars and mineral salts which are quite attractive substrates for biotechnological processes. (Nitschke and Pastore 2003) tested the five cassava flour wastewater (manipueira) preparations as culture media for biosurfactant production by a wild-type *Bacillus s*p. isolate. Growth and biosurfactant production was seen in all preparations. In a subsequent study (Nitschke et al. 2004) applied two *Bacillus subtilis *strains for biosurfactant production on cassava effluent as a substrate. Both *B. subtilis *ATCC 21332 and *B. subtilis *LB5a, exhibited good surface activity and produced similar yields of surfactin.

The same group evaluated a combinatorial approach for biosurfactant production by some bacterial isolates using molasses, milk whey and cassava flour wastewater and compared their production with the production on conventional medium (Nitschke and Pastore 2004). Initial studies indicated many isolates were able to grow and exhibit excellent surface activity when supplemented with Manipueira medium. In an attempt to broaden the substrate stocks for economic production of biosurfactants (Nitschke and Pastore 2006) investigated the production and properties of a biosurfactant, synthesized by *Bacillus subtilis *LB5a strain, using cassava wastewater as substrate. The crude surfactant (a lipopeptide) with concentration of 3.0 g/l could withstand exposure to elevated temperatures (100°C), high salinity (20% NaCl) and a wide range of pH values and formed stable emulsions with various hydrocarbons. They concluded that cassava wastewater was a suitable substrate for biosurfactant biosynthesis. Barros et al. (2008) reported the production of biosurfactant by *Bacillus subtilis *LB5a on a pilot scale using cassava wastewater as the substrate. The study was carried out using heated clear cassava wastewater in a 40-liter batch pilot bioreactor adapted for simultaneous foam collection during the fermentative process. Biosurfactant was precipitated from the foam to yield 2.4 g/l and had good surface activity (surface tension of 27 mN/m and the critical micellar concentration of 11 mg/l).

These studies demonstrated that starch rich substrates and waste materials can be used as a substrate for the production of biosurfactants due to its nutrients contents such as carbohydrates, metallic ions, nitrogen and others that make nutritional supplementation unnecessary.

## Other unconventional substrate sources

There are few studies carried out with some renewable substrates mainly confined to a particular geographic region. (Deshpande and Daniels 1995) used the abundantly available, inexpensive animal's fats to investigate sophorolipids production by *C. bombicola *and achieved 120 g/l of sophorolipids. (George and Jayachandran 2008) reported the use of orange fruit peeling as sole carbon source for rhamnolipid production using *P. aeruginosa *MTCC 2297. Citrus fruits are one of the most important value added fruit crop in international market and is mostly used for orange juice production which generates large quantities of waste (Adalgisa et al. 2005).

(de Gusmão et al. 2010) studied biosurfactant production by *Candida glabrata *using vegetable fat waste as substrate. They applied a factorial design to investigate the effects and interactions of waste, yeast extract and glucose on the surface tension after 144 h cultivation. Maximum surface activity was achieved with vegetable fat waste at 5% and yeast extract at 0.2%. The biosurfactant containing cell-free broth retained its surface-active properties after incubation at high temperatures, at a wide range of pH values and salt concentrations. Structural determination suggests it to be a mixture of carbohydrates, proteins and lipids and the authors further concluded its suitability for use in bioremediation and oil recovery. This was the first report on the use of a vegetable fat waste as substrate for the production of a biosurfactant.

Another attractive substrate which has found use for production of biosurfactants is the byproduct of Cashew industry which is important in Brazil. Cashew apples are rich in reducing sugar, vitamins and minerals salts and are cheap (US $ 0.50/kg) which makes them an interesting and inexpensive culture medium (Campos et al. 2002; Honorato et al. 2007; Rabelo et al. 2009). (Rocha et al. 2007) evaluated the ability of *Pseudomonas aeruginosa *to produce biosurfactants using cashew apple juice (CAJ) and mineral media supplemented with peptone and nutritive broth. Reduction in surface tension of the medium indicated that CAJ could be used as medium for growth and biosurfactant production. Another group from Brazil reported the utilization of mineral medium containing clarified cashew apple juice (MM-CAJC) by *Bacillus subtilis *LAMI008 strain (Ponte Rocha et al. 2009). In various combination of supplementation they showed that the highest reduction in surface tension was achieved with the cultivation on MM-CAJC, supplemented with yeast extract. The produced biosurfactant (surfactin) exhibited good surface and emulsifying activity and a yield of 3.5 mg/L was obtained when MM-CAJC, supplemented with yeast extract, was used. This was a second successful feasibility study, to produce surfactin from clarified cashew apple juice. Similar work but with a different *Bacillus subtilis *LAMI005 strain was reported by (Giro et al. 2009). They reported surfactin yield of 123 mg/l of clarified cashew apple juice supplemented with mineral medium (MM-CCAJ). Biosurfactant produced showed good surface activity and emulsifying ability asserting the fact that it was feasible to produce surfactin from CCAJ, a renewable and low-cost carbon source.

Biosurfactant production has been reported using Okara in Japan. Okara is the residue left from ground soy beans after extraction of the water extractable fraction used to produce soy milk and *tofu*. It is an industrial waste and disposed of mostly by incineration (O'Toole 1999). Approximately 700,000 tons of okara are produced annually from the production of tofu in Japan, (Kuan and Liong 2008). About 1.1 kg of fresh okara is produced from every kilogram of soybeans processed for soy milk. It is composed of water (81.1%), protein (4.8%), fat (3.6%), starch and sugar (6.4%), fiber (3.3%), and ash (0.8%). (Ohno and coworkers (1993a), b, 1995, 1996) reported the utilization of okara for the simultaneous production of a lipopeptide surfactin and iturin in solid state fermentation (SSF) by *Bacillus subtilis *NB22.

Researchers have utilized simple alcohol glycerol for biosurfactant production. Glycerol is the principal by-product obtained during transesterification of vegetable oils and animal fats (da Silva et al.2009). Glycerol being a component of lipids is abundant in nature. Many known microorganisms are capable of naturally utilizing the glycerol as a sole carbon and energy source. Glycerol usually serves as a substitute for common carbohydrates, such as sucrose, glucose and starch (Bognolo 1999). Recent surge in biodiesel production has led to increased accumulation of glycerol as byproduct of this industry. The low cost glycerol could be used as water soluble substrate for biosurfactant production. (Nitschke et al. 2005b) reported the utilization of glycerol as sole carbon source by *Pseudomonas aeruginosa *for synthesis of rhamnolipid. Although yields were less compared to traditional hydrophobic substrates but cheap substrates can overcome the yield drawbacks (Rahman et al. 2002b) reported 1.77 g/l rhamnolipid biosurfactant production by *P. aeruginosa *DS10-129. In another study involving the glycerol as substrate Zhang et al. (Zhang et al. 2005) produced 15.4 g/l rhamnolipids using *P. aeruginosa *growing on a basal mineral medium containing glycerol as the sole carbon source. These studies clearly demonstrate the feasibility of utilizing glycerol as carbon source for growth and biosurfactant production by microbes. (Morita et al. 2007) showed that a basidiomycete yeast, *Pseudozyma antarctica *JCM 10317, efficiently produced mannosylerythritol lipids (MELs) as glycolipid biosurfactants from glycerol. The amount of MEL yield reached 16.3 g/l by intermittent feeding of glycerol.

(da Silva et al. 2009) used mineral medium formulated with glycerol (93%) for biosurfactant production by *Pseudomonas aeruginosa *UCP0992. They achieved a yield of 8.0 g/l after 96 h with surface tension reduction to 27.4 mN/m. High emulsification index (E 24) value of 80% and CMC of 700 mg/l was obtained after 72 h of growth. The study is noteworthy for possible biosurfactant production from glycerol with potential of application in the environment. Liu et al. (Liu et al., 2011) applied a known glycolipid producer *Ustilago maydis *to efficiently convert biodiesel-derived crude glycerol to glycolipids. This study suggests that *U. maydis *is an excellent host for the bioconversion of crude glycerol to value-added products.

(Lee et al. 2004) reported the use of fish oil for biosurfactant production. They optimized the culture medium for the *Pseudomonas aeruginosa *BYK-2 KCTC 18012P for enhanced rhamnolipids production and used 25 g/l fish oil as carbon source. In optimum conditions, they achieved a yield of 17 g/l of rhamnolipid. This was a unique report of use of fish oil as substrate for biosurfactant production.

These studies clearly indicate the vast potential of the unconventional substrates for the biosurfactant production. Most of these substrates are low cost economical substrates and will help economize the biosurfactant production.

## Biosurfactant coproduction with renewable substrate

The above sections highlighted ways to contribute towards the reduction in cost of the starting substrate to cut down the overall biosurfactant production process. Another interesting approach for achieving more fruitful results will be co-production of biosurfactants and other important metabolites. There are reports of such coproduced metabolites e.g. polyhydroxyalkanotes (PHA), lactic acid and other metabolites.

### a. Biosurfactants and PHA

*Pseudomonas aeruginosa *produces rhamnolipids and PHA. PHA have been applied for manufacture of bottles, films and fibers as an biodegradable packaging agent (Sudesh et al. 2000). (Füchtenbusch et al. 2000) attempted the co-production approach for PHA production using the remaining oil from rhamnolipid production. During screening for the bacteria which can use residual oil from biotechnological rhamnose production as a carbon source for growth they identified *Ralstonia eutropha *H16 and *Pseudomonas oleovorans *capable of using this waste material as the sole carbon source for growth and production of PHA. The approach adopted reduced the PHA production cost by using the remaining carbon source for rhamnolipid production. (Hori et al. 2002) demonstrated the feasibility of the simultaneous production of PHAs and rhamnolipids, as a novel approach to reduce their production costs, by the cultivation of *Pseudomonas aeruginosa *IFO3924. Large yields of PHAs and rhamnolipids were obtained using decanoate as a better carbon source than ethanol and glucose for the simultaneous production. (Costa et al. 2009) evaluated glycerol, cassava wastewater, waste cooking oil an and cassava waste with waste frying oils as alternative low-cost carbon substrates for the production of rhamnolipids and PHA by various *Pseudomonas aeruginosa *strains. Cassava waste with frying oil was best substrate for the overall production of rhamnolipids and PHAs. The study demonstrated the feasibility of use of cassava waste with frying oil as an alternate and economical substrate for dual production of biosurfactants and PHAs.

In another study by (Marsudi et al. 2008) demonstrated palm oil can be directly utilized for the simultaneous production of polyhydroxyalkanoates (PHAs) and rhamnolipids using *Pseudomonas aeruginosa *IFO3924. Using secreted lipase for palm oil hydrolysis they showed consumption of fatty acids as carbon sources for PHAs production and glycerol for rhamnolipid production. Both PHA and rhamnolipid production was nitrogen dependent and occurred in nitrogen limited conditions. In their recent work (Hori et al. 2011) investigated the effects of temperature and carbon length of fatty acid substrates on the simultaneous production of PHA and rhamnolipids by *P. aeruginosa *IFO3924. Differential temperature optimum obtained for PHA (30°C) and rhamnolipid syntheses (28°C) suggests that the product ratio between these two products can be controlled by changing temperature.

This approach for simultaneous production for rhamnolipid and PHAs by using whole or part of the available substrates makes the process economically viable and attractive.

### b. Biosurfactants and Proteases

Microbial proteases especially alkaline proteases are an important groups of industrial enzymes that cater to the requirement of nearly 60% of the world enzyme market (Gupta et al. 2002). These proteases have numerous industrial applications involving detergents, food, leather, silk, waste management and pharmaceuticals (Gupta et al. 2002). However, the single biggest market of their use is in the detergent formulations. Most of these enzyme productions are confined to genus *Bacillus *which is known to be a common biosurfactant producer (Kim et al.. 1997; Makkar and Cameotra 1998; Makkar and Cameotra 1999). With many complementary properties such as excellent detergency, emulsifying, foaming and dispersing traits to the proteases a concomitant usage of biosurfactant and protease could offer improved efficacy as detergent additives. (Ramnani et al. 2005) showed the concomitant production of protease and biosurfactant using cornstarch and soy flour as carbon and nitrogen sources. They achieved an overall 2.3-fold increase for both protease (2954 U/ml) and biosurfactant (41%). They used ultrafiltration (100 kDa cutoff) as a cost-effective purification of both protease and biosurfactant where the surfactant traps the protease on the membrane thereby detaining both in the retenate. The dry product mixture with sodium sulfate was stable with a year shelf life.

These studies signify the ecomomicity of concomitant production of biosurfactant with other metabolite thus easing off the cost factor for the overall biosurfactant production.

## Conclusions

Surfactants are an important class of chemical products in view of the volumes sold and of their great variety of applications. Biosurfactants and surfactants derived from renewable raw materials are progressively entering into the market. Net economic gains in lieu with the production costs and applicability will be the determining factors for use of renewable materials for the production of biosurfactants. Possible links between the production conditions of these molecules, their structure and functions are paramount factors to optimize the strategic view of their potential industrial development. The production of biosurfactants with high added-value properties is the central part of future research. Considering their vast potential for large field of applications, their development needs broad cooperation across disciplines in order to fully characterize and identify their potential uses. New value adding opportunities will result from the identification of specific applications of biosurfactants in relation to their biological applications as antibiotic, antifungal, insecticide, antiviral and antitumor agent. Their use as immunomodulators, enzyme inhibitors or in high cost product will help developing newer applications for them.

The application of economical technologies and process based on utilization of waste conversion to products is also gaining ground. The commercial realization of the biosurfactants which is restricted by the high production costs can be equipoise by optimized production conditions provided by utilization of the cheaper renewable substrates and application of novel and efficient multistep downstream processing methods. Recombinant and mutant hyper-producer microbial strains, able to grow on a wide range of cheap substrates may produce biosurfactants in high yield and potentially bring the required breakthrough for their economic production (Banat et al. 2010).

This effort is global as seen in results of utilization of the local based waste as molasses in India, oil based wastes in South America, potato and potato based wastes in USA. In future, the creation of database for agricultural substrates will help to document the range different compositions and quality of substrates and their influence on the biosurfactants' types and purity. This would involve the selection of suitable strains with the desired properties, use of inexpensive alternative substrates, application of a factorial design approach for optimizing process parameters, and enhancing yields. The cumulative enhancements of each process step will make substantial progress towards an economical technology. The true significance of these processes will be justified only when these studies will be scaled up to commercially viable processes.

## Competing interests

The authors declare that they have no competing interests.
